# Termite Fungus Comb Polysaccharides Alleviate Hyperglycemia and Hyperlipidemia in Type 2 Diabetic Mice by Regulating Hepatic Glucose/Lipid Metabolism and the Gut Microbiota

**DOI:** 10.3390/ijms25137430

**Published:** 2024-07-06

**Authors:** Haihan Xiao, Xudong Song, Peng Wang, Weilin Li, Senhua Qin, Chaofu Huang, Beimin Wu, Bao Jia, Qionghua Gao, Ziyi Song

**Affiliations:** 1 Guangxi Key Laboratory of Animal Breeding, Disease Control and Prevention, College of Animal Science and Technology, Guangxi University, Nanning 530004, China; 2 Nanning Institute of Termite Control, Nanning 530023, China; 3 Guangxi Key Laboratory of Agri-Environmental and Agri-Products Safety, National Demonstration Center for Experimental Plant Science Education, College of Agriculture, Guangxi University, Nanning 530004, China

**Keywords:** termite fungus comb, polysaccharides, type 2 diabetes, glucose metabolism, lipid metabolism, gut microbiota

## Abstract

Type 2 diabetes (T2D) is a chronic metabolic disorder characterized by hyperglycemia and dyslipidemia. The termite fungus comb is an integral component of nests of termites, which are a global pest. Termite fungus comb polysaccharides (TFCPs) have been identified to possess antioxidant, anti-aging, and immune-enhancing properties. However, their physicochemical characteristics and their role in fighting diabetes have not been previously reported. In the current study, TFCPs were isolated and structurally characterized. The yield of TFCPs was determined to be 2.76%, and it was found to be composed of a diverse array of polysaccharides with varying molecular weights. The hypoglycemic and hypolipidemic effects of TFCPs, as well as their potential mechanisms of action, were investigated in a T2D mouse model. The results demonstrated that oral administration of TFCPs could alleviate fasting blood glucose levels, insulin resistance, hyperlipidemia, and the dysfunction of pancreatic islets in T2D mice. In terms of mechanisms, the TFCPs enhanced hepatic glycogenesis and glycolysis while inhibiting gluconeogenesis. Additionally, the TFCPs suppressed hepatic de novo lipogenesis and promoted fatty acid oxidation. Furthermore, the TFCPs altered the composition of the gut microbiota in the T2D mice, increasing the abundance of beneficial bacteria such as *Allobaculum* and *Faecalibaculum*, while reducing the levels of pathogens like *Mailhella* and *Acetatifactor*. Overall, these findings suggest that TFCPs may exert anti-diabetic effects by regulating hepatic glucose and lipid metabolism and the composition of the gut microbiota. These findings suggest that TFCPs can be used as a promising functional ingredient for the prevention and treatment of T2D.

## 1. Introduction

Diabetes is a metabolic disorder characterized by persistently elevated blood glucose levels. Long-term hyperglycemia contributes significantly to the development of various chronic diseases and complications, including hyperlipidemia, inflammation, and damage to vital organs such as the kidneys, eyes, heart, and blood vessels [1]. Consequently, diabetes has emerged as a major and intractable global public health challenge [1]. Among the various types of diabetes, type 2 diabetes (T2D) is the most prevalent, accounting for approximately 90% of all diabetes cases worldwide [2]. A pivotal factor leading to T2D is insulin resistance, which is often triggered by unhealthy eating habits, obesity, and genetic predisposition. Current strategies for managing T2D primarily involve the administration of oral hypoglycemic agents, encompassing biguanides, sulfonylureas, glucagon-like peptide-1 (GLP-1) receptor agonists, insulin sensitizers, α-glucosidase inhibitors, and thiazolidinediones [3]. However, these medications are often accompanied by a range of undesirable side effects like diarrhea and nausea [4,5]. Because of that, the search for alternative hypoglycemic approaches that are both low in toxicity and high in efficacy, to either aid in the treatment of diabetes or alleviate its symptoms, has garnered increasing attention.

Traditional Chinese medicines (TCMs) emerge as a promising alternative treatment for T2D, due to their multi-target mechanisms, fewer side effects, and relatively lower costs [6]. For example, herbal extracts from *Scutellaria baicalensis* [7,8], *Coptis chinensis* [9,10], *Pueraria lobata* [11,12], *Zingiber officinale* [13], and *Panax notoginseng* [14] have shown positive outcomes in clinical practice for managing T2D. Nevertheless, our current understanding of the therapeutic potential of TCM in treating T2D remains incomplete, necessitating further investigation.

The termite fungus comb, a critical component within the nests of termites, is primarily composed of termite feces, food remnants, and a specific array of fungi capable of decomposing lignocellulose, thereby providing nutritional sustenance for the termites [15]. This fungus comb has been traditionally used as a medicine among the Zhuang people. According to the records in “Zhuang Medicine of China”, the termite fungus comb exhibits cough-suppressing and asthma-relieving properties, and is commonly employed to treat symptoms such as chronic cough, chronic bronchitis, and asthma. In recent years, studies have underscored the efficacy of termite fungus comb extracts in exerting antimicrobial, anti-aging, and immunostimulatory effects [16,17]. It is noteworthy that the primary constituents of the termite fungus comb are carbohydrates [18]. Additionally, polysaccharides, a type of carbohydrate, have been found to possess anti-diabetic properties [19]. However, there is a notable lack of reports investigating the physicochemical characterization of termite fungus comb polysaccharides (TFCPs), as well as their hypoglycemic and hypolipidemic effects.

Hence, this work was designed to prepare and characterize TFCPs, and explore the effects and underlying mechanisms of oral TFCPs against T2D in mice from the perspective of hepatic glucose and lipid metabolism as well as the modification of gut microbiota. The findings will offer new insights for the development of innovative drugs for the treatment of T2D.

## 2. Results

### 2.1. Extraction and Characterization of TFCPs

**Figure 1:**
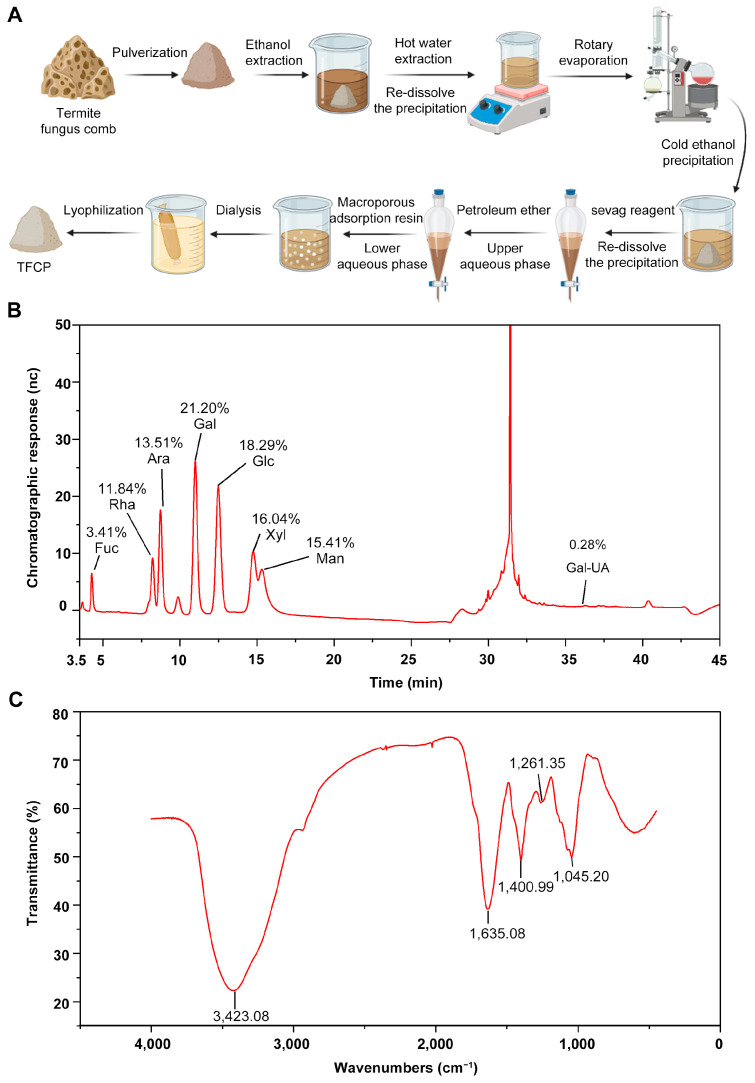
Extraction and characterization of TFCPs. (**A**) Process of TFCP extraction (created in BioRender.com). (**B**) Monosaccharide composition of TFCPs according to ion chromatogram analysis. (**C**) FT-IR spectrum of TFCPs in the range of 4000–450 cm^−1^.

The extraction of TFCPs was achieved through stepwise ethanol precipitation from termite fungus combs following the protocol presented in Figure 1A. Then, the physicochemical characterization of these TFCPs was analyzed. Initially, the molecular weight and uniformity of the TFCPs were evaluated using high-performance gel permeation chromatography (HPGPC). The results, presented in Table 1, indicate that TFCPs have an average molecular weight (Mn) of 23.593 kDa, a peak molecular weight (Mp) of 17.923 kDa, a weight-averaged molecular weight (Mw) of 58.917 kDa, a z-averaged molecular weight (Mz) of 1452.528 kDa, and a polydispersity index (Mw/Mn) of 2.497. These data indicate that the molecular weight distribution of TFCPs is relatively dispersed, suggesting that TFCPs are a mixture composed of polysaccharide molecules of various sizes.

Subsequently, the monosaccharide composition of the TFCPs was determined using ion chromatography. This analysis revealed that the TFCPs were primarily composed of fucose (3.41%), rhamnose (11.84%), arabinose (13.53%), galactose (21.2%), glucose (18.29%), xylose (16.04%), mannose (15.41%), and galacturonic acid (0.28%) (Figure 1B), indicating TFCPs have a complex structure (Figure 1B). To further investigate the functional groups of TFCPs, Fourier-transform infrared spectroscopy (FT-IR) was employed. The resulting spectrum revealed several important peaks, including a strong absorption peak at 3423.08 cm^−1^, which is indicative of O-H stretching vibrations (Figure 1C). Additional peaks were observed at 1045.2 cm^−1^ (C-O stretching vibration) [20], 1400.99 cm^−1^ (C-H bending vibration), and 1261.35 cm^−1^ (C-O-C stretching vibration) (Figure 1C). These peaks are typical features of sugar molecules, indicating the presence of sugar units in the polysaccharides. Additionally, a significant peak was observed at 1635.08 cm^−1^, suggesting the presence of C=O stretching vibrations, typical of glycosides [21], further demonstrating the presence of sugar units connected by glycosidic bonds in the polysaccharides (Figure 1C).

### 2.2. TFCPs Improve Systemic Glucose and Lipid Homeostasis in T2D Mice

**Figure 2:**
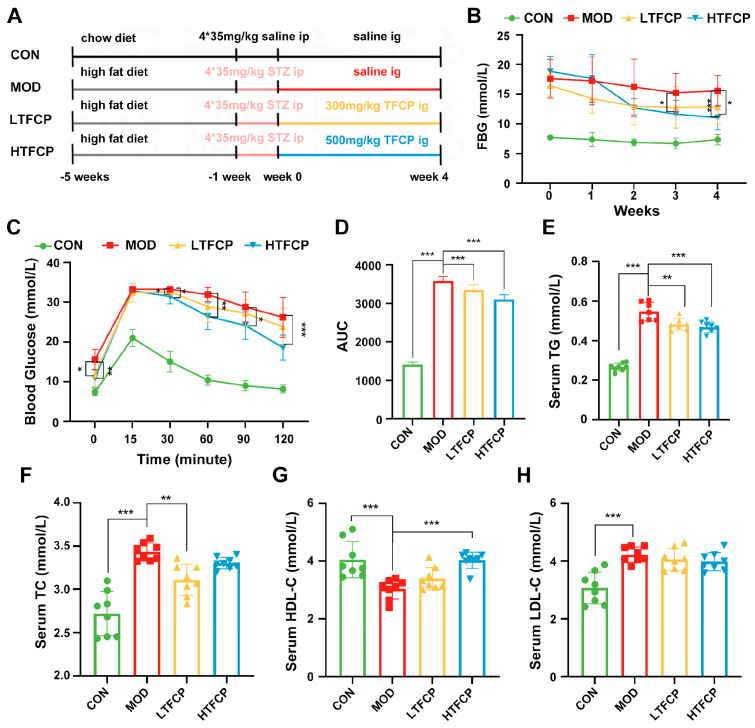
Effects of TFCPs on systemic glucose and lipid homeostasis. (**A**) Animal experimental protocol (drawing by Figuredraw v2.0). (**B**) Fasting blood glucose levels of each group during the experimental period. (**C**) Blood glucose levels during oral glucose-tolerance test at week 4. (**D**) Area under the curve of the OGTT. (**E**–**H**) Serum TG, TC, LDL-C, and HDL-C levels. Data are presented as the mean ± SD. * *p* < 0.05, ** *p* < 0.01, *** *p* < 0.001; *n* = 8.

Next, to assess the potential anti-diabetic benefits of TFCPs in mice, we designed an experimental protocol as illustrated in Figure 2A. Our initial step was to confirm the successful establishment of a T2D mouse model. As depicted in Figure 2B, the mice subjected to a high-fat diet and streptozotocin (STZ) injection exhibited significantly elevated fasting blood glucose (FBG) levels compared to the control group, confirming the induction of T2D in these mice. Subsequently, we observed that the administration of TFCPs at high doses (500 mg/kg) for 3 weeks significantly reduced FBG levels. Similarly, after 4 weeks of treatment with lower doses of TFCPs (300 mg/kg), a significant improvement in FBG levels was noted. These findings indicate that TFCPs are capable of effectively alleviating elevated FBG levels in T2D mice. In line with these observations, oral glucose-tolerance tests (OGTT) further revealed that TFCPs enhance systemic glucose metabolism, particularly at high doses (Figure 2C,D). To assess the impact of TFCPs on lipid metabolism, we analyzed the blood lipid profile. Consistent with previous findings [22], the diabetic mice showed increased levels of triglycerides (TGs), total cholesterol (TC), and low-density lipoprotein cholesterol (L-LDL), as well as decreased levels of high-density lipoprotein cholesterol (H-LDL), when compared to the control group (Figure 2E–H). However, TFCP treatment partially reversed these alterations in the blood lipid profile (Figure 2E–H), suggesting that TFCPs can improve lipid metabolism in a T2D mouse model. Taken together, these data indicate that TFCPs hold the potential to prevent hyperglycemia and hyperlipidemia resulting from T2D, effectively diminishing the likelihood of developing diabetic complications.

### 2.3. TFCPs Ameliorate the Morphology and Function of Pancreatic Islets in T2D Mice

**Figure 3:**
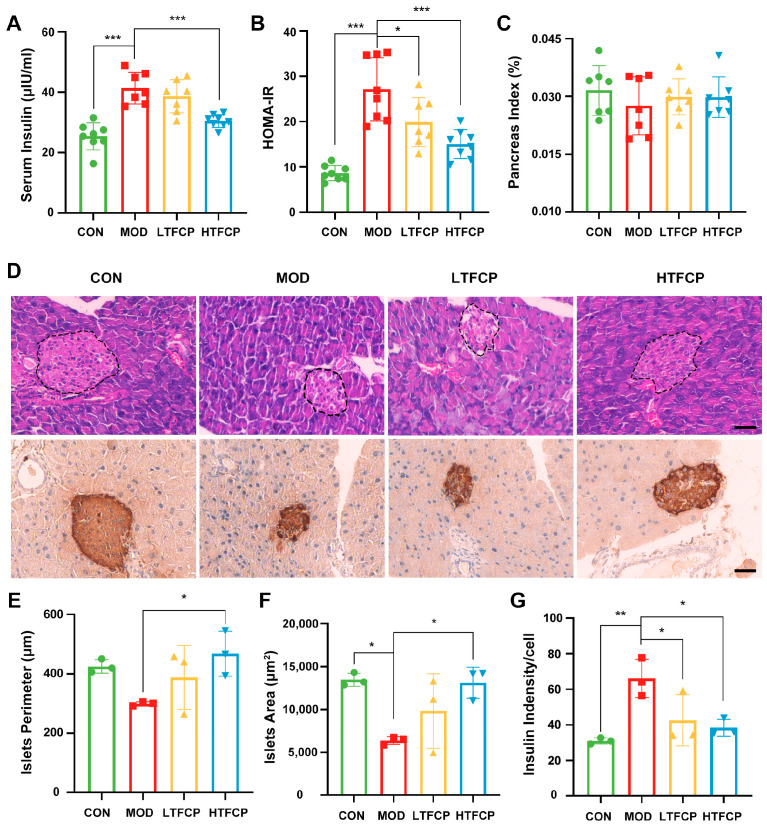
Effects of TFCPs on islet morphology and function. (**A**,**B**) Serum insulin levels and HOMA-IR after 4 h of fasting at week 4 (n = 7–8). (**C**) Pancreas index (n = 7–8). (**D**) Insulin IHC and H&E staining of the pancreas (scale bar = 40 μm). (**E**,**F**) Analysis of pancreatic islet perimeter and area based on H&E staining (n = 3). (**G**) Analysis of average insulin density per cell based on IHC staining (n = 3). Data are presented as the mean ± SD. * *p* < 0.05, ** *p* < 0.01, *** *p* < 0.001.

Pancreatic islets play a pivotal role in maintaining systemic glucose homeostasis by secreting insulin, yet their functionality is significantly impaired under the condition of T2D [23]. Thus, we assessed whether the morphology and function of the pancreatic islets in diabetic mice was recovered after TFCP treatment. As shown in Figure 3A,B, the diabetic mice exhibited elevated serum insulin levels and significant insulin resistance when compared to the control group. However, treatment with TFCPs in the diabetic mice resulted in a reduction in serum insulin levels and a marked improvement in insulin resistance. Subsequently, we evaluated the pancreatic islets’ functionality in the mice. While there was no change in the overall mass of the pancreas among the different groups (Figure 3C), substantial differences in the pancreatic islets were observed through insulin immunohistochemical staining (Figure 3D). Specifically, the pancreatic islets in the diabetic mice displayed atrophy, disorganization, and vacuolar degeneration. In contrast, the islets in the high-TFCP-treated group exhibited a more regular morphology, distinct margins, and the absence of apparent lesions, resembling the state observed in the control group (Figure 3D). Further histological analysis revealed that the diabetic mice had smaller islets with a higher insulin density per cell compared to the control group. Interestingly, TFCP treatment led to an increase in islet size and a decrease in insulin density per cell (Figure 3E–G). These compelling findings indicate that TFCPs may exert a favorable influence on the restoration of dysfunctional pancreatic islets in diabetic mice, thus providing a protective effect against the progression of T2D.

### 2.4. TFCPs Enhance Hepatic Glucose Metabolism in T2D Mice

**Figure 4:**
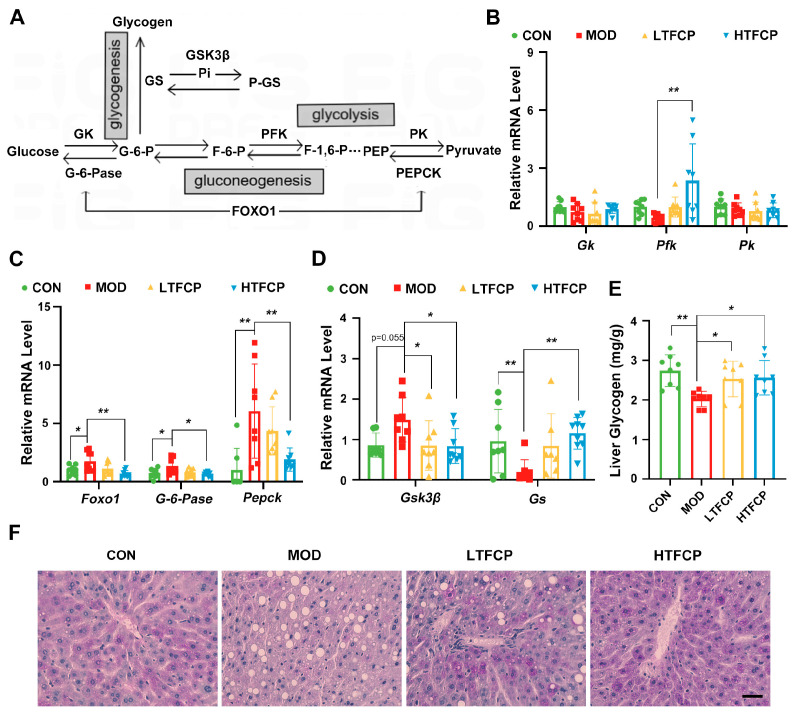
Effects of TFCPs on hepatic glucose metabolism. (**A**) Schematic representation of downstream signaling pathways in glycolysis, gluconeogenesis, and glycogenesis in hepatocytes. (**B**–**D**) qRT-PCR analysis of glucose-metabolism-related genes in the liver, as depicted in (**A**). (**E**) Liver glycogen levels. (**F**) PAS staining of the liver (scale bar = 20 μm). Data are presented as the mean ± SD. * *p* < 0.05, ** *p* < 0.01; *n* = 8.

Subsequently, our focus shifted to the liver, given its pivotal role in regulating systemic glucose metabolism via pathways such as glycolysis, gluconeogenesis, and glycogen synthesis (Figure 4A). Initially, we examined the impact of TFCPs on the expression of key glycolytic genes involved in glycolysis. The results indicated that high-dose TFCP treatment significantly increased the levels of phosphofructokinase (*Pfk*), which had been downregulated in the diabetic mice, while showing no effect on glucose kinase (*Gk*) and pyruvate kinase (*Pk*) expression (Figure 4B). This outcome suggests that TFCP treatment enhances hepatic glycolysis. Subsequent to this, the expression of key gluconeogenic genes was assessed. Our findings revealed that high-dose TFCP treatment notably attenuated the upregulation in mRNA levels of forkhead box O1 (*FoxO1*), glucose-6-phosphatase (*G6Pase*), and phosphoenolpyruvate carboxykinase (*Pepck*) that is typically observed in the diabetic state (Figure 4C), indicating that TFCP administration hinders hepatic gluconeogenesis. Lastly, we investigated the effects of TFCPs on the expression of genes encoding enzymes crucial for glycogen synthesis. The results demonstrated that the TFCPs suppressed the expression of glycogen synthase kinase 3β (*Gsk3β*), an inhibitor of glycogen synthesis, while promoting the expression of glycogen synthase (*Gs*), which facilitates glycogen synthesis (Figure 4D). Consistent with the gene expression data, TFCP treatment significantly elevated the hepatic glycogen content in the diabetic mice (Figure 4E,F), suggesting that TFCP treatment enhances the capacity for glycogen synthesis. In summary, treatment with TFCPs significantly improves hepatic glucose metabolism by enhancing glycolysis and glycogen synthesis, while effectively inhibiting gluconeogenesis.

### 2.5. TFCPs Boost Hepatic Lipid Metabolism in T2D Mice

**Figure 5:**
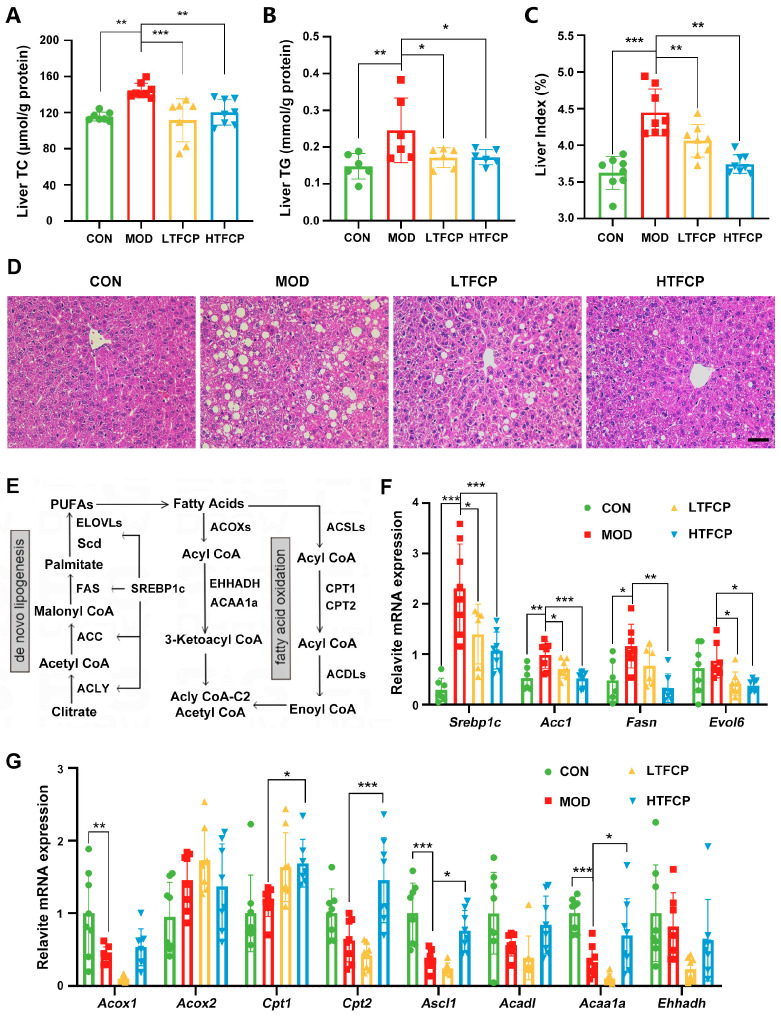
Effects of TFCPs on hepatic lipid metabolism. (**A**,**B**) Liver TG and TC levels (n = 5–8). (**C**) Liver index. (**D**) H&E staining of the liver (scale bar = 25 μm). (**E**) Schematic representation of downstream signaling pathways in de novo lipogenesis and fatty acid oxidation in hepatocytes. (**F**,**G**) qRT-PCR analysis of lipid-metabolism-related genes in the liver, as depicted in (**E**). Data are presented as the mean ± SD. * *p* < 0.05, ** *p* < 0.01, *** *p* < 0.001; *n* = 8.

Next, we explored the mechanisms by which TFCPs alleviate lipid disorders in T2D mice. Given the vital role of liver lipid metabolism in maintaining overall lipid homeostasis in the body, our focus narrowed to examining the impact of TFCPs on hepatic lipid metabolism. Initially, we observed that TFCP administration significantly attenuated the elevations in hepatic TG and TC levels, as well as the liver index, which were induced in the diabetic mice (Figure 5A–C). Moreover, histological examination results concurred with these findings, revealing a marked reduction in lipid droplet accumulation in the livers of the diabetic mice treated with both low and high doses of TFCPs (Figure 5D). These data suggest that TFCPs can effectively ameliorate hepatic lipid disorders in T2D mice.

The body’s hepatic lipid content is tightly regulated by a balance between two primary processes: de novo lipogenesis and fatty acid β-oxidation (Figure 5E). To elucidate the molecular mechanisms underlying TFCPs’ impact on hepatic lipid metabolism, we assessed their effects on the expression of genes encoding enzymes involved in both lipid catabolism and anabolism. Our results showed that the expression of key lipogenic genes, including sterol regulatory element-binding transcription factor 1 (*Srebp1c*), acetyl-CoA carboxylase (*Acc1*), fatty acid synthase (*Fasn*), and ELOVL fatty acid elongase 6 (*Evol6*), was upregulated in the diabetic mice compared to the control mice, and this upregulation was significantly repressed after TFCP treatment (Figure 5F). In contrast, TFCP treatment induced a reversal of the downregulated expression observed in type 2 diabetic conditions for genes involved in lipid oxidation, such as carnitine palmitoyltransferase 1/2 (*Cpt1/2*), acyl-CoA synthetase long-chain family member 1 (*Acsl1*), and acetyl-CoA acyltransferase 1A (*Acaa1a*) (Figure 5G). Collectively, these findings indicate that TFCPs effectively alleviate hepatic lipid overload by suppressing lipogenic gene expression and promoting the expression of genes involved in lipid oxidation.

### 2.6. TFCPs Optimize the Diversity and Composition of Gut Microbiota in T2D Mice

**Figure 6:**
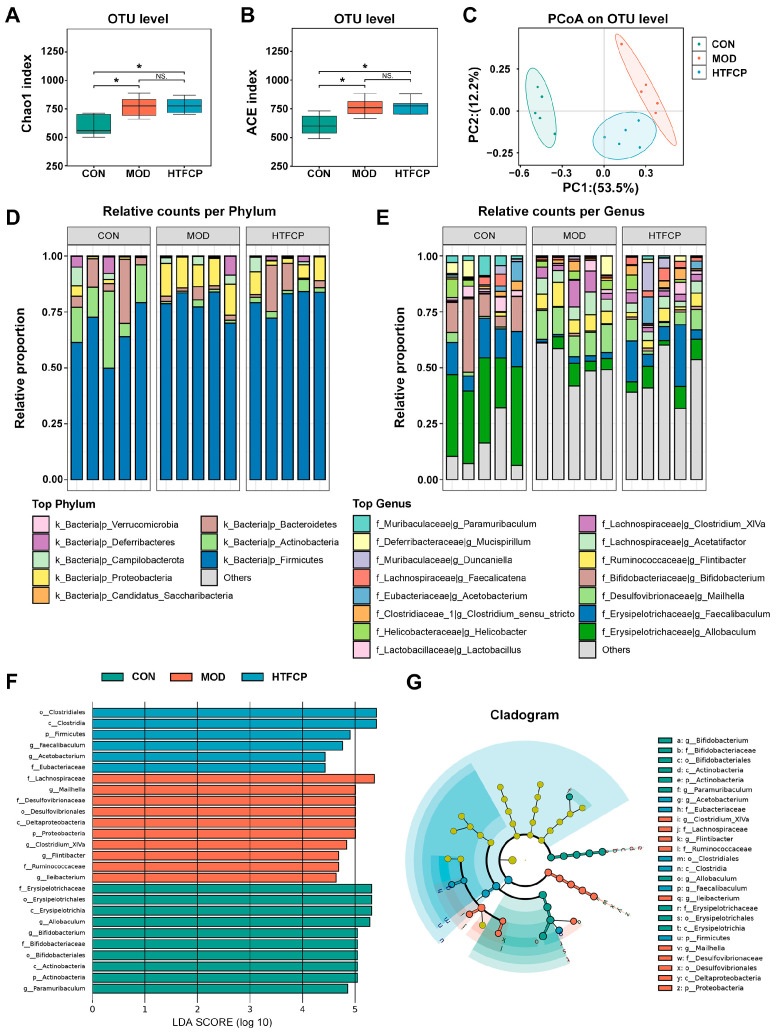
Effects of TFCPs on the diversity and composition of gut microbiota. (**A**,**B**) Alpha diversity analysis. (**C**) PCoA of OTU levels. (**D**) Microbiota composition analysis at the phylum level. (**E**) Microbiota composition analysis at the genus level. (**F**,**G**) Linear discriminant analysis (LDA) effect size (LEfSe) analysis of OTU levels (only taxa with LDA scores of more than 3 are presented). Data are presented as the mean ± SD. ns stands for not significant,* *p* < 0.05, *n* = 5.

Recent studies have shed light on the fact that, in addition to that in the liver, an imbalance in microbial communities in the gut constitutes a significant contributor to the dysregulation of host glucose and lipid metabolism [24]. To explore this further, we conducted a comparative analysis of the diversity and composition of gut microbiota among the different groups. Our findings revealed that the diabetic mice exhibited an increased diversity and richness of gut microbiota compared to the control mice. However, HTFCP administration did not alter the overall diversity of the gut microbiota of the diabetic mice (Figure 6A,B). A UniFrac principal coordinate analysis (PCoA) of β-diversity, on the other hand, revealed a distinct separation between the diabetic mice and control mice, with the HTFCP-treated mice clustering closer to the control mice (Figure 6C). This indicates that TFCP treatment may have remodeling effects on the composition of gut microbiota.

We further investigated the specific changes in the gut microbiota composition at the phylum and genus levels. At the phylum level, we observed a significant increase in the abundance of Firmicutes and Proteobacteria, and a decrease in Bacteroidetes, in the diabetic mice compared to those observed in the control mice. TFCP treatment, however, significantly reduced the abundance of Proteobacteria and increased that of Bacteroidetes, with no significant effect on the abundance of Firmicutes (Figure 6D). At the genus level, the abundance of *Allobaculum* and *Faecalibaculum* was significantly reduced in the diabetic mice compared to that in the control mice, but was significantly increased following HTFCP intervention. Additionally, the abundance of *Mailhella*, *Flintibacter*, *Acetatifactor*, and *Clostridium_XlVa* was significantly increased in the diabetic mice compared to that in the control mice, and decreased after TFCP intervention.

Finally, we employed the linear discriminant analysis effect size (LEfSe) method to identify discriminative microbes at different taxonomic levels. At the genus level, *Allobaculum*, *Bifidobacterium*, and *Paramuribaculum* were dominant in the control mice, while *Mailhella*, *Clostridium_XlVa,* and *Flintibacter* were the most differentially abundant taxa in the diabetic mice. In contrast, *Faecalibaculum* and *Acetobacterium* were the predominant genera in the HTFCP-treated mice (Figure 6F,G). In conclusion, our data suggest that the diabetic condition significantly disrupts the composition of the gut microbiota, and TFCP treatment partially restores the balance of the gut microbiota in T2D mice.

### 2.7. TFCPs Modify the Function of Gut Microbiota in T2D Mice

**Figure 7:**
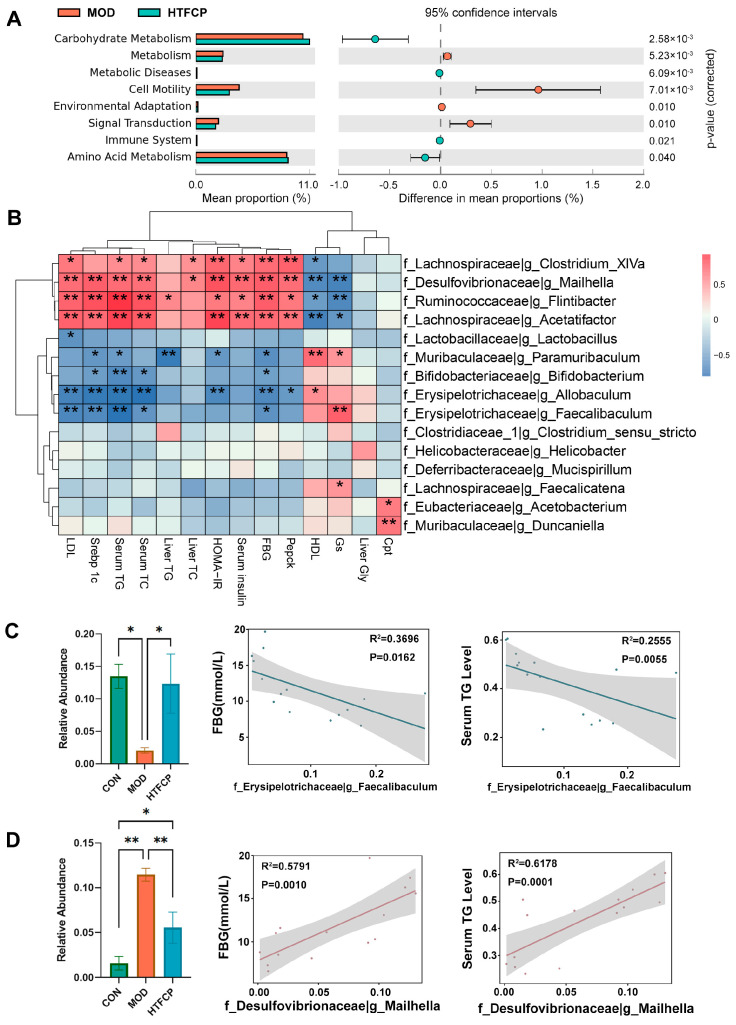
Correlation analysis between key OTUs and T2D-related biochemical indicators. (**A**) PICRUSt2 analysis of KEGG pathways. (**B**) Heatmap showing correlation analyses between T2D-related biochemical indicators and the relative abundances of gut microbiota at the genus level, showcasing the top 20 microorganisms. (**C**,**D**) Alterations in the relative abundance of Faecalibaculum or Mailhella and their correlation with FBG and serum TG levels. Data are presented as the mean ± SD. * *p* < 0.05, ** *p* < 0.01; *n* = 5.

To further explore the potential functional interactions between gut microbiota and host glucose and lipid metabolism, we employed the Phylogenetic Investigation of Communities by Reconstruction of Unobserved States (PICRUSt) algorithm to perform a Kyoto Encyclopedia of Genes and Genomes (KEGG) functional analysis. As depicted in Figure 7A, the carbohydrate metabolism pathways were notably enriched in the HTFCP-treated mice compared to those observed in the control mice, indicating that TFCP administration enhances the microbiota’s function in glucose metabolism. Supporting this finding, we conducted a Pearson correlation analysis, which further elucidated the strong associations between intestinal gut flora and metabolic indexes (Figure 7B). Specifically, *Faecalibaculum* [25], a genus that was downregulated in the diabetic mice but upregulated in the HTFCP-treated mice, demonstrated a negative correlation with the levels of FBG and serum TGs (Figure 7C). Conversely, *Mailhella* [26], a genus that was upregulated in the diabetic mice but downregulated in the HTFCP-treated mice, showed a positive correlation with the levels of FBG and serum TGs (Figure 7D). These data collectively suggest that gut microbial genera may play a role in the TFCP-induced improvement of glucose and lipid metabolism in T2D mice.

## 3. Discussion

Termites, as eusocial arthropods, serve as decomposers that contribute to enhanced soil fertility and increased crop yields [27]. Nevertheless, certain termite species are posing a growing threat to agriculture, inflicting significant direct and indirect damage on the agricultural system. Global economic losses due to termites are estimated to exceed USD 40 billion (United States dollars) per year, prompting extensive research into strategies for managing and controlling termite populations [28]. Termite fungus combs, a crucial element within termite nests, play a vital role in the survival and reproduction of these insects. In this study, we have discovered that polysaccharides isolated from termite fungus combs are composed of a diverse array of polysaccharides with varying molecular weights. Furthermore, these polysaccharides have been found to possess the potential for inducing anti-diabetic effects. Thus, this study not only expands our understanding of the medicinal value of termite fungus combs, but also holds significant importance for advancing termite control through promoting their medical utilization.

It is well known that in T2D, peripheral insulin resistance triggers a compensatory response in the pancreatic islets, particularly the β-cells, resulting in an augmented secretion of insulin to meet the body’s elevated glucose demands. Nonetheless, this compensatory hyperinsulinemia fails to effectively regulate blood glucose levels due to the presence of insulin resistance. Prolonged hyperglycemia, coupled with insulin resistance, can culminate in β-cell dysfunction and apoptosis, resulting from mechanisms such as glucotoxicity, thereby further exacerbating metabolic dysregulation and compromising glycemic control [29]. In accordance with conventional understanding, the current study observed that the model mice exhibited hallmark phenotypic manifestations of T2D, encompassing elevated levels of fasting blood glucose and serum insulin, alongside a reduction in pancreatic islet size. Notably, the administration of TFCPs was able to partially reverse these pathological alterations, thereby suggesting their efficacy in alleviating the symptoms associated with T2D.

The liver serves as a central hub for various metabolic processes, as it gathers the nutrient flow drained from the gut through the portal vein [30]. These processes encompass carbohydrate metabolism and the maintenance of lipid homeostasis. The metabolic processes involved in carbohydrate metabolism primarily include glycolysis, glycogenesis, and gluconeogenesis, whereas those involved in lipid metabolism primarily include lipogenesis and fatty acid oxidation [31]. These processes are critical for maintaining whole-body energy homeostasis in response to nutrient levels and physiological status, and any disruption of these processes can have systemic consequences, such as obesity, insulin resistance, fatty liver disease, and T2D [30]. Consequently, we explored the underlying mechanisms of TFCPs’ anti-diabetic effects, particularly from the perspective of hepatic glucose and lipid metabolism. Consistent with the dogmas of the situation in T2D, the livers from the diabetic mice displayed disturbances in their glucose and lipid handling, manifesting as increased lipid accumulation and a reduced glycogen content. However, TFCP treatment could rectify these disturbances, not only in the phenotypes but also in the expression of key enzymes. Although its exact mechanisms are still unknown, studies have identified adenosine 5′ monophosphate-activated protein kinase (AMPK) as a major upstream metabolic hub that is involved in mediating the biological effects of polysaccharides [32,33]. Therefore, future studies could further investigate the direct effects of TFCPs on AMPK-mediated signaling pathway.

A wealth of research has established the critical role of gut microbiota in maintaining host glucose and lipid homeostasis through the production of gut metabolites, such as short-chain fatty acids (SCFAs) [34,35,36]. Thus, to further understand the mechanisms behind TFCPs’ anti-diabetic effects, we conducted an investigation focusing on the role of the gut microbiota. Surprisingly, we found that the α-diversity of gut microbiota was increased, rather than decreased, in diabetic mice compared with that in the control mice (Figure 6A,B). This discrepancy could be attributed to differences in our experimental design since the gut microbiota is a complex and dynamic ecosystem that is influenced by a wide range of factors, including diet, lifestyle, genetics, and disease status. Therefore, the diet composition used in our study may have had a differential effect on the microbiota compared to what has been observed in previous studies. Additionally, the duration of the diabetes induction may also have influenced the microbiota’s diversity, as changes in microbiota tend to occur gradually over time. In fact, this unexpected phenomenon has also been previously reported [25,37]. Moreover, our findings indicate that TFCPs do not significantly alter the α-diversity of the intestinal microbiota (Figure 6A,B). Curiously, the literature presents inconsistent reports on the influence of polysaccharides on α-diversity. For instance, polysaccharides extracted from moringa oleifera leaves [38] have been shown to augment the α-diversity of the gut microbiota in mice fed a high-fat diet. Conversely, crude guava polysaccharides and oyster polysaccharides [39,40] have been reported to have no significant effects on α-diversity. These discrepancies may arise from variations in the composition and dosage of the polysaccharides used. Despite this, our β-diversity analysis revealed significant differences among the three groups studied. At the phylum level, Firmicutes and Bacteroidetes were the most predominant, with previous studies suggesting that a lower Firmicutes/Bacteroidetes ratio is associated with a reduced risk of obesity [41]. In our study, the diabetic mice exhibited an increased Firmicutes/Bacteroidetes ratio compared to the control mice. However, TFCP treatment, by increasing Bacteroidetes, partially restored this ratio, indicating a more balanced microbiota community. At the genus level, the most significant changes were observed in *Allobaculum*. *Allobaculum*, a member of the Firmicutes phylum, has been identified as a short-chain fatty acid-producing bacterium [42,43]. We found that the abundance of *Allobaculum* was reduced in the diabetic mice, but this was slightly reversed with HTFCP treatment. Furthermore, our correlation analysis revealed that the abundance of *Allobaculum* was associated negatively with indexes of lipid profiles and insulin resistance, and positively with the level of serum HDL, suggesting its potential as an anti-obesity biomarker. Our findings align with those of multiple previous studies [37,44,45,46], although a few have reported increased *Allobaculum* abundance with high-fat feeding [47,48]. Such inconsistencies may have resulted from differences in the experimental design and 16s rRNA analysis methods. Additionally, we identified *Faecalibaculum* as another bacterium that decreased under diabetic conditions and increased with TFCP treatment. The abundance of *Faecalibaculum* was correlated negatively with the levels of serum LDL, serum TGs, serum TC, FBG, and hepatic *Srebp1c*, and positively with the level of hepatic *Gs*, suggesting its beneficial role in glucose and lipid metabolism. Wang et al. have also reported similar findings [25], further supporting the notion that *Faecalibaculum* is a beneficial bacterium with regard to T2D treatment. Moreover, we found that genera such as *Mailhella*, *Acetatifactor*, *Flintibacter*, and *Clostridium_XlVa* were increased in the diabetic mice and decreased following TFCP treatment. Our observations regarding *Mailhella* and *Acetatifactor* are supported by previous studies [26,39,49]. However, for *Flintibacter*, current research is limited, warranting further investigation. In contrast, our findings on *Clostridium_XlVa* contradict the results of one previous study [39], which may be due to the use of different mouse models: our study employed a T2D mouse model, whereas this previous study used a high-fat-diet-induced obese mouse model.

However, it is worth noting that an accumulating body of research suggests that polysaccharides can regulate glucose and lipid metabolism through the gut–liver axis [50,51]. Consequently, dietary TFCPs may initially reshape the intestinal microbiota, subsequently promoting the production of SCFAs and other favorable metabolites. These microbial metabolites, once absorbed by the intestine, are partially metabolized by intestinal cells for energy. The remaining portion then enters the liver via the portal circulation. In the liver, SCFAs directly activate AMPK by elevating the AMP/ATP ratio [52]. Consequently, activated AMPK elicits various beneficial metabolic effects, including the suppression of hepatic glucose production and fatty acid synthesis, as well as the promotion of hepatic glycolysis and fatty acid oxidation [53]. Therefore, TFCPs may also alleviate hyperglycemia and hyperlipidemia in T2D mice by modulating the intestinal microbiota-related gut–liver axis.

## 4. Materials and Methods

### 4.1. TFCP Preparation

The termite fungus combs were collected from a chestnut field in Long’an County, Nanning City, Guangxi Zhuang Autonomous Region, China. The termites have been identified by Jia Bao, the Director of the Scientific Research Department of the Nanning Termite Control Institute, as belonging to *Macrotermes annandalei* (Silvestri). The TCFPs were extracted as previously described with minor modifications [54,55,56]. Briefly, the termite fungus combs were dried at 45 °C to a constant weight and pulverized into powder with 60-mesh sieves. The termite fungus comb powder was added to 4 times its volume of absolute alcohol, followed by extracting with stirring for 3 h to remove the fat-soluble pigment and some impurities. The mixture was then centrifuged at 6000× *g* for 10 min to collect the precipitation. Pure water was added to the precipitation until the concentration reached 100 g/L, and then it was extracted using a 60 °C water bath for 4 h, and centrifuged. The supernatant was collected, and the residue was extracted twice following the steps mentioned above. The two extracts were combined and concentrated to 1/10 of their original volume using a rotary evaporator, then added with four times the volume of anhydrous ethanol at 4 °C overnight and centrifuged at 8000× *g* for 10 min to collect the crude polysaccharide precipitation. 

This precipitation was fully dissolved in pure water, and enzymatically hydrolyzed with 0.2–0.6‰ papain overnight. Then, a 1/4 mixture volume of savage reagent (chloroform/n-butanol = 4:1, *v*/*v*) was added into the mixture with thorough shaking. The upper aqueous phase was collected, a 1/4 mixture volume of petroleum ether was added with thorough mixing, and then the lower aqueous phase was collected. An equal volume of macroporous resin AB-8 was added to the aqueous phase, thoroughly mixed, and adsorbed overnight. The liquid was collected, and small molecular components were removed via dialysis in a 3 KDa dialysis bag for 48 h. Finally, the pure polysaccharides (yield: 2.76%; purity: 53.9%) were obtained using a freeze dryer and designated as the TFCPs. The TFCP extraction workflow used in this study is illustrated in Figure 1A.

### 4.2. Chemical Analysis of TFCPs

A 100 mg amount of lyophilized TFCPs was sealed in a centrifuge tube and sent to Sanshu Biotechnology (Nantong, China) for testing. The molecular weight of these TFCPs was analyzed with high-performance gel permeation chromatography (HPGPC). The column temperature was maintained at 45 °C, with an injection volume of 100 μL. The mobile phase consisted of 0.02% NaN_3_ and 0.1 M NaNO_3_, and the flow rate was set at 0.5 mL/min. The elution gradient was conducted for 100 min. Monosaccharide components were analyzed using ion chromatography (IC, ICS 5000+, Thermo Fisher Scientific, Sunnyvale, CA, USA) and a liquid chromatography column (10 μm, 150 × 3.0 mm, DIONEX^TM^ CarboPac^TM^ PA20, Sunnyvale, CA, USA). The functional group of TFCPs was determined using a Fourier-transform infrared spectrometer (FT-IR, Nicolet iZ-10, Thermo Fisher Scientific, Waltham, MA, USA). A small quantity of the polysaccharide sample was weighed and mixed with 200 mg of potassium bromide (KBr), then pressed into a 1 mm thick pellet for scanning.

### 4.3. Animal Studies and Experimental Protocols

All animal studies were conducted in accordance with protocols approved by the Animal Ethics Committee of Guangxi University (Gxu-2022-343). Thirty-two male C57BL/6J mice (weight: 20 ± 2 g; 3 weeks old) were purchased from Guangxi Medical University (Nanning, China) and reared in specific pathogen-free facilities under controlled lighting and temperature conditions (12 h light/dark cycle; 22 ± 2 °C). After one week of adaptation, these 32 mice were divided into a control group (CON; n = 8) and an experimental group (n = 24). The control group received a normal diet throughout the experiment, while the experimental group was fed a high-fat diet. After 4 weeks, the experimental-group mice were intraperitoneally injected with 35 mg/kg of streptozotocin (dissolved in 0.1 mol/L sodium citrate buffer to obtain a 10 mg/mL solution) for four consecutive days. Three days after the last injection, their fasting blood glucose (FBG) levels were measured, and mice with FBG levels greater than 11.1 were designated as T2D mice. All T2D mice were randomly divided into three groups: the T2D mouse model group (MOD; distilled water), the group treated with a lower dose of TFCPs (LTFCP; 300 mg/kg TFCP), and the group treated with a higher dose of TFCPs (HTFCP; 500 mg/kg TFCP). They were orally administered the respective treatments via gavage for 4 weeks, and their FBG levels were measured weekly.

### 4.4. OGTT

At the fourth week of TFCP intervention, an OGTT (1.5 g/kg, 20% glucose solution) was performed in mice that had fasted for 15 h, and the blood glucose of these mice was measured at 0, 15, 30, 60, 90, and 120 min after glucose administration.

### 4.5. Biochemical Assay

Serum fasting insulin levels were measured using an insulin enzyme-linked immunosorbent assay kit (JL11459-96T, Shanghai Jianglai Biotechnology Co., Ltd., Shanghai, China); serum and liver triglycerides (TGs), total cholesterol (TC), high-density lipoprotein cholesterol (HDL-C), and low-density lipoprotein cholesterol (LDL-C) were measured using various assay kits (A110-1-1, A111-1-1, A112-1-1, and A113-1-1, Nanjing Jiancheng Institute of Bioengineering, Nanjing, China); and liver glycogen was measured using a glycogen-content detection kit (BC0345, Solarbio Life Science, Beijing, China). All data were measured with an ultra-micro ultraviolet spectrophotometer (Multiskan SkyHigh, Thermo Fisher Scientific, Waltham, MA, USA).

### 4.6. Histological Examinations

The pancreas and liver were immersion-fixed in 4% paraformaldehyde for 24 h, after which they were processed by Wuhan Sevier Biotechnology Co., Ltd. (Wuhan, China) for embedding, sectioning, and staining. This included hematoxylin and eosin (H&E) staining, periodic acid–Schiff (PAS) staining for glycogen, and immunohistochemistry (IHC) analysis for insulin. The images were collected under a light microscope (Biological microscope ML31; MSHOT, Guangzhou, China), and the cell area and density were quantified using Image J software (v1.46r).

### 4.7. RNA Extraction and qRT-PCR

Total RNA was extracted following our previous method [57]. It was then reverse-transcribed using Recombinant Ribonuclease Inhibitor (2313A, Takara Biomedical Technology Co., Ltd., Beijing, China) and M-MLV Reverse Transcriptase (M1701, Promega Corporation, Beijing, China) to synthesize cDNA synthesis. The cDNA was added with a 2× premixed real-time fluorescence quantitative rapid PCR mixture (A301-01, GenStar, Beijing, China) for qRT-PCR testing. The primer sequences used in this RT-qPCR testing are provided in Table S1. 

### 4.8. Fecal Microbiome Analysis

Fresh fecal samples were collected and sent to Suzh PANOMIX Biomedical Tech Co., LTD (Suzhou, China) for sequencing. The V3 and V4 (a) regions of the bacteria 16S were amplified using the following primers: F-ACTCCTACGGGA GGCAGCA and R-GGACTACHVGGGTWTCTAAT. The reaction system had a volume of 25 μL, and the amplification parameters were as follows: initial denaturation at 98 °C for 2 min, followed by 25–30 cycles of denaturation at 98 °C for 15 s, annealing at 55 °C for 30 s, and extension at 72 °C for 30 s, with a final extension at 72 °C for 5 min, and then holding at 4 °C.

16S rDNA sequencing analysis was performed according to previous methods [58]. Sequence files were processed using QIIME2 (v2022.8) for initial processing, including quality filtering and demultiplexing. The data were then denoised using the DADA2 module to generate an Amplicon Sequence Variant (ASV) table. Subsequently, the ASV table was normalized to 10,000 feature sequences per sample to ensure comparability across the samples for downstream analysis. Using the GreenGenes (https://greengenes.lbl.gov, accessed on 15 May 2023) reference database, the 16S rDNA genes were annotated through a naive Bayes classifier. Alpha diversity, gauged by Chao1 and ACE indices, served to evaluate the intricacy of species diversity, and these indices were computed using the Vegan package (v2.6-4, R package). Beta diversity, assessed through Bray–Curtis distance matrix, was visualized via principal coordinate analysis (PCoA) to explore nuances in the sample community compositions. Taxonomic abundances at the phylum and genus levels were summarized using Phyloseq (v1.46.0, R package). Differential-genera bacteria were discerned employing LEfSe analysis. PICRUSt analysis was performed using ImageGP (https://www.bic.ac.cn/BIC/#/, accessed on 15 October 2023), and the results were visualized with STAMP software (v2.1.3). Pearson correlation analysis was used to show the correlation between bacterial abundance and T2D-related biochemical indicators. The visualization of all findings was achieved using the ggplot2 package (v3.4.1) within the R environment.

### 4.9. Data Analysis

All data were processed using GraphPad Prism 9.0., and the multiple groups were assessed with an ordinary one-way ANOVA analysis of variance for comparison. All data are presented as the mean ± SD, and statistical significance was determined when *p* < 0.05.

## 5. Conclusions

In conclusion, our findings indicate that TFCPs are composed of a diverse array of polysaccharides with varying molecular weights. Oral administration of TFCPs significantly improved hyperglycemia and hyperlipidemia in our T2D mice, as evidenced by reduced levels of FBG, serum insulin, and serum lipids, as well as improved glucose tolerance and pancreatic islet function. Mechanistically, TFCP treatment enhanced liver glucose and lipid metabolism and optimized the composition of the gut microbiota. These results suggest that TFCPs may potentially be explored as an effective functional ingredient for the management of T2D. However, the detailed mechanisms underlying these effects warrant further investigation.

## Figures and Tables

**Table 1:** Gel permeation chromatography analysis of TFCPs.

	Mn	Mp	Mw	Mz	Mw/Mn
(kDa)	23.593	17.923	58.917	1452.528	2.497

## Data Availability

The data of this study will be made available in the BioProject dataset at http://www.ncbi.nlm.nih.gov/bioproject/1086696, accessed on 12 March 2024 with the reference ID PRJNA1086696.

## Supplementary Materials

The following supporting information can be downloaded at https://www.mdpi.com/article/10.3390/ijms25137430/s1.
