# Supplementary material for: Termite Fungus Comb Polysaccharides Alleviate Hyperglycemia and Hyperlipidemia in Type 2 Diabetic Mice by Regulating Hepatic Glucose/Lipid Metabolism and the Gut Microbiota
Source: Int J Mol Sci. 2024 Jul 6;25(13):7430. doi: 10.3390/ijms25137430 (PMC11242180; doi:10.3390/ijms25137430)
Supplement: Supplementary file 1 [file ijms-25-07430-s001.zip › ijms-3074331-supplementary.pdf]

Table S1. qRT-PCR primers.

Gene	GeneID	Forward Primer (5'-3')	Reverse Primer (5'-3')
β-actin	XM_021187106.2	CACGATGGAGGGGCCGGACTCATC	TAAAGACCTCTATGCCAACACAGT
Gs	XM_021213686.2	AGGACATTTCAGGGATTAA	GCCATCCATCTCCATCTGC
Gsk3β	XM_021209356.2	CACCTGCACTCTTCAACTTTAC	CACGGTCTCCAGCATTAGTATC
FoxO1	XM_021158151.2	TATGTCACCGGTTGATCCCG	TTATGAGATGCCTGGCTGCC
Glut2	XM_021196507.2	GCTGTCTCTGTGCTGCTTGT	CGTAACTCATCCAGGCGAAT
Gk	XM_063242828.1	AAAACACGTATGGAACAGGGTG	TTAGCCAGCGGATTACAGCA
Pfk	NM_008826.5	ACCGTGGACCTGGAGAAA	AGCCCTGACAGCAGCATT
Pk	NM_001378867.1	TAGTTCTCACGGAGTCTGGC	*CAGCACGGCATCCTTACA*
G-6-Pase	XM_021194719.1	TCTTCCTGTTTGCCCTCG	TCGGCTTGGTGCCATTTT
Pepck	XM_021194801.2	TCATCATCACCCAAGAGCA	CCACCACATAGGGCGAGT
Srebp1c	XM_030245748.1	AGGAGGACATCTTGCTGCTTCT	GATCTCTGCCAGTGTTGCCATG
Acc1	XM_030245463.1	CTGGAGCTAAACCAGCACTCCCGAT	GAGCTGACGGAGGCTGGTGACA
Fasn	XM_030245556.1	CACTGCATTGACGGCCGGGT	GGACAAGCCCAGGCTGCGAG
Elovl6	XM_029537282.1	TCAACGAGAACGAAGCCATCCA	TTCATCAGATGCCGACCACCAA
Acox1	NM_001377522.1	GCCAAGGCGACCTGAGTGAGC	ACCGCAAGCCATCCGACATTC
Acox2	XM_036158940.1	ACGGTCCTGAACGCATTTATG	TTGGCCCCATTTAGCAATCTG
Cpt1	XM_029547870.1	GAACACAAATGTGCAAGCAGC	GCCATGACCGGCTTGATCTC
Cpt2	NM_009949.3	CAGCACAGCATCGTACCCA	TCCCAATGCCGTTCTCAAAAT
Acsl1	NM_001302163.2	CGATGGCTGTTGGACTTTGC	CACCCAGGCTCGACTGTATC
Acad1	XM_021158666.2	TCTTTTCCTCGGAGCATGACA	GACCTCTCTACTCACTTCTCCAG
Acaa1a	NM_001357516.1	AGGCTTCAAGAACACCACCC	GGCTCCTGGCTCAAGAACAT
Ehhadh	NM_023737.3	CGGTCAATGCCATCAGTCCAA	TGCTCCACAGATCACTATGGC
